# Supplementary material for: Mechanistic role of pyroptosis in Kawasaki disease: An integrative bioinformatics analysis of immune dysregulation, machine learning-based biomarker discovery, WGCNA, and drug repurposing insights
Source: PLoS One. 2025 May 14;20(5):e0323597. doi: 10.1371/journal.pone.0323597 (PMC12077800; doi:10.1371/journal.pone.0323597)
Supplement: Attachment — Submitted filename: Response to Reviewers.docx [file pone.0323597.s002.docx]

**Academic Editor's Comment 1:
The authors failed to introduce that COVID and variants of concerns can cause KD. The authors should introduce this.**

**Author Response:
Thank you for this important suggestion. In response, we have updated the Introduction section to include a brief discussion regarding the association between COVID-19 (and its variants) and Kawasaki disease (KD). Relevant literature (Liu et al., 2021; Liu et al., 2025) has also been cited accordingly.**

**Academic Editor's Comment 2:**

The authors should introduce the function of Myd88. Pattern recognition receptors, such as Myd88, play important roles in recognizing viral nucleic acids as a major class of viral pathogen-associated molecular pattern and triggering intra cellular signaling pathways that culminate in activation of interferon regulatory factor 3 (IRF3) and NF-κB. More references should be cited, with this one (PMID: 38556084) as an example (citing is optional)

**Author Response:**

**We appreciate this valuable comment. In response, we have revised the Discussion section to include a brief introduction to the biological function of MYD88, particularly its role in TLR and IL-1R signaling pathways, which activate downstream IRF3 and NF-κB transcription factors. We have also cited the recommended reference (PMID: 38556084) to support this addition. The revision is located in the Discussion section.**

**Reviewer #1 Comment 1:**

**What is the relationship between MYD88/S100A12 and Kawasaki disease in previous studies? Is there any literature review? In other words, how do the authors looking at the simulation findings if they are similar to the findings of previous studies?**

**Author Response:**

**Thank you for this valuable suggestion. We have revised the Discussion section to include a brief literature review on the previously reported roles of MYD88 and S100A12 in Kawasaki disease. Specifically, we cited studies demonstrating their upregulation in KD and their involvement in inflammatory and immune responses. We also discussed the consistency between our findings and prior research, which supports the reliability and biological relevance of our analysis.**

****Reviewer #1 Comment 2:****

**Results 7, ‘MYD88 showed significant positive correlations with M1 macrophages, activated NK cells, and regulatory T cells (Tregs)’, does not match Figure 7C, please check.**

****Author Response:****

**Thank you for pointing out this discrepancy. We have carefully reviewed Figure 7C and the corresponding Results section. The sentence has been corrected to ensure consistency with the figure. The revised statement now accurately reflects the data presented in Figure 7C.**

****Reviewer #1 Comment 3:****

**Results 7, ‘S100A12 exhibited significant positive correlations with M1 macrophages, regulatory T cells (Tregs), activated NK cells, and CD8 T cells, and negative correlations with CD4 naïve T cells’, does not match Figure 7D, please check.**

****Author Response:****

**Thank you for noting this discrepancy. We have carefully reviewed Figure 7D and the corresponding text. The statement in the Results section has been corrected to accurately reflect the correlation patterns shown in Figure 7D. We apologize for the oversight and have updated the manuscript accordingly.**

****Reviewer #1 Comment 4:****

**Figure 7A, ‘Immune Cell’, ‘activated’ is incomplete, please check.**

****Author Response:****

**Thank you for pointing out this issue. We have reviewed Figure 7A and found that the label for “Immune Cell”and "activated" was incomplete. We have corrected the label in the revised figure to ensure it is fully displayed and properly aligned with the rest of the content.**

****Reviewer #1 Comment 5:****

**Figure 7B, ‘cKD’, ‘healthy’ is incomplete, please check.**

****Author Response:****

**Thank you for pointing out this issue. We have reviewed Figure 7B and found that the label for ‘cKD’ and ‘healthy’ was incomplete. We have corrected the label in the revised figure to ensure it is fully displayed and properly aligned with the rest of the content.**

****Reviewer #2 Comment 1:****

**The data used is transcriptomic, so I would prefer to see protein-level expression of S100A12 and MYD88, either from wet lab confirmation, such as Western blotting or other database.**

****Author Response:****

**We greatly appreciate this valuable suggestion. We acknowledge that confirming expression changes at the protein level would enhance the validity of our findings. However, due to the retrospective nature of our study and current limitations in obtaining suitable biological samples, we were unable to perform wet-lab protein validation such as Western blotting. We agree this is an important next step, and we have clearly stated this limitation and suggested further protein-level validations in future studies in our manuscript.**

****Reviewer #2 Comment 2:****

**From clinical study perceptive, The sample information can be briefly described especially the clinical characteristics. These factors may contribute the expression profile difference. And more sample and diversity data are also encouraged.**

****Author Response:****

**Thank you for highlighting this important point. We fully agree that detailed clinical information and increased sample diversity would enhance the interpretation of expression differences. Unfortunately, due to the publicly available datasets used in this analysis, we are limited to the clinical data provided by the original authors, which is relatively brief. We have clearly acknowledged this limitation in the revised manuscript and recommended that future studies include detailed clinical information and a broader range of samples to validate our findings further.**

****Reviewer #2 Comment 3:****

**From a biological perspective, I believe it would be valuable to include expression profiles from KD patients who received IVIG treatment (a common therapy) as part of the validation. It would be interesting to observe whether the protein levels of S100A12 and MYD88 change in response to treatment.**

****Author Response:****

**Thank you very much for this constructive suggestion. In response, we conducted an additional validation using transcriptomic data (dataset GSE16797) from IVIG-responsive KD patients. The results demonstrated significant downregulation of both MYD88 and S100A12 expression after IVIG treatment (Figure 5E and 5F), supporting their involvement in KD inflammation and their potential as markers for treatment response. Although protein-level validation remains an important future direction, our current transcript-level validation supports the biological relevance of these genes in the treatment response context.**

****Reviewer #2 Comment 4:****

**Figure 1G could further clarify the different portions—specifically, which are DEGs, which are genes retrieved from databases, and which are identified through your analysis. The label "pyroptosis" is too close to the WGCNA module; it would be better if the font style matched that of Figure 4G.**

****Author Response:****

**Thank you for the helpful suggestion. We have updated Figure 1G to clearly label the different portions of the Venn diagram. The section representing differentially expressed genes (DEGs) is now labeled accordingly, as well as the sections for genes retrieved from external databases and novel genes identified through our analysis. These clarifications should improve the figure’s clarity and interpretation.**

****Reviewer #2 Comment 5:****

**Figure 3C: For the Y-axis title, would “expression level” be more appropriate?**

****Author Response:****
**Thank you for your suggestion. We have updated the Y-axis title in Figure 3C to “Expression level” to improve clarity and better reflect the data being presented. We believe this change enhances the figure's readability.**

****Editor Comment（2025-04-03）:****
**Please confirm at this time whether or not your submission contains all raw data required to replicate the results of your study. Authors must share the “minimal data set” for their submission. PLOS defines the minimal data set to consist of the data required to replicate all study findings reported in the article, as well as related metadata and methods (https://journals.plos.org/plosone/s/data-availability#loc-minimal-data-set-definition).**

****Author Response:****
**Thank you for your comment. To comply with the data sharing policy, we have uploaded t**he raw gene expression data supporting the findings of this study are publicly available and can be accessed through the following sources: [https://github.com/wangchen-0214/Kawasaki-Pyroptosis-dataexpr.git](https://github.com/wangchen-0214/Kawasaki-Pyroptosis-dataexpr.git). DOI :10.5281/zenodo.15129021.

The code used for data analysis in this study is publicly available at <https://github.com/wangchen-0214/Kawasaki-Pyroptosis.git.> DOI: 10.5281/zenodo.15125948.
